# Supplementary material for: Design of a hazard prediction system with intelligent multimodal fusion based on artificial intelligence & internet of things technology: taking a crib as an example
Source: PeerJ Comput Sci. 2024 Oct 29;10:e2404. doi: 10.7717/peerj-cs.2404 (PMC11623082; doi:10.7717/peerj-cs.2404)
Supplement: Supplemental Information 4 [file peerj-cs-10-2404-s004.pdf]

Design of a hazard prediction system with intelligent multimodal fusion based on AIoT technology: Taking a crib as an example

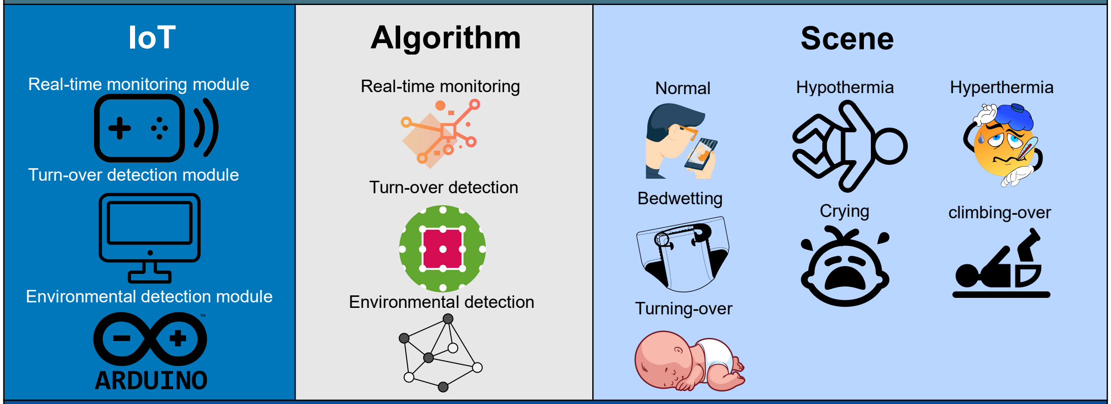

Conclusion: The article presents an AIoT-based intelligent hazard prediction system that utilizes an IoT hardware device and AI algorithm to detect real-time status and predict potential dangers. The system demonstrates transferability and can be applied to various scenarios, including baby cribs, infants and elderly patient care in the hospital, and abandoned baby care stations.
